# Vibrational spectroscopic characterisation of fluorescent-protein-tagged and wild-type bacteria in surface-associated microdroplets

**DOI:** 10.1039/d5fd00175g

**Published:** 2026-04-20

**Authors:** Cassio Lima, Jontana Allkja, Rasmita Raval

**Affiliations:** a Open Innovation Hub for Antimicrobial Surfaces, Surface Science Research Centre, Department of Chemistry, University of Liverpool Liverpool L69 3BX UK raval@liverpool.ac.uk

## Abstract

Fluorescent protein (FP) tagging is widely used to differentiate bacterial populations in mixed-species and surface-associated systems. However, FP expression can impose a metabolic burden and alter cellular physiology, raising concerns about whether FP-tagged strains reliably represent their wild-type counterparts. Understanding the biochemical consequences of FP expression is therefore essential when interpreting data from fluorescence-based studies. In this study, we employed Fourier-transform infrared (FTIR) microscopy and Raman confocal microscopy to evaluate the impact of FP tagging in Gram-positive *Staphylococcus aureus* and Gram-negative *Pseudomonas aeruginosa* labelled with eGFP- and mCherry-fluorescent proteins, respectively. Principal component analysis (PCA) of FTIR spectra revealed clear species-level discrimination driven by differences in lipid, protein, polysaccharide, and phosphate content characteristic of Gram-positive and Gram-negative bacteria. Importantly, no detectable spectral differences were observed between wild-type and FP-expressing strains. Raman spectroscopy offered stronger species-specific contrast owing to resonance enhancement of carotenoids in *S. aureus* and cytochromes in *P. aeruginosa*. PCA applied to Raman spectra from GFP-labelled *Staphylococcus aureus* strain and its wild-type counterpart again showed no difference. Together, these results demonstrate that FP expression under the experimental conditions used in this study did not induce detectable changes in vibrational spectral datasets, thus enabling species-level differentiation to be retained in the presence of FP tagging. This paves the way forward for correlative fluorescence and vibrational mapping of bacterial populations at surfaces.

## Abstract 2

Vibrational spectroscopy combined with PCA does not reveal biochemical differences between fluorescent-protein-tagged and wild-type bacteria in surface-associated microdroplets.

## Graphical abstract

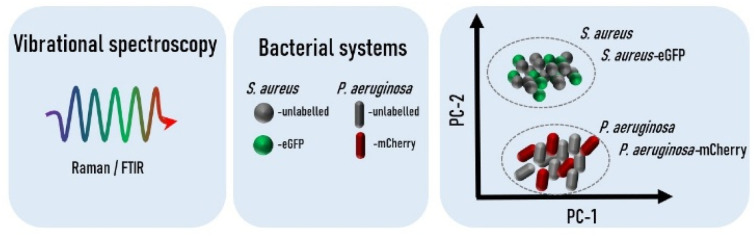

## Introduction

Globally, bacterial infections remain a leading cause of mortality, contributing to approximately 4.95 million deaths in 2019, of which 1.27 million were directly attributable to antimicrobial resistance (AMR).^1^ In many natural, clinical, and built environments, bacteria are frequently encountered as micron-scale droplets deposited on surfaces, arising from processes such as coughing, sneezing, speaking, or environmental aerosolization.^2,3^ These surface-associated droplets play a critical role in microbial transmission and persistence, particularly in healthcare and community settings, where they can act as reservoirs for infection and cross-contamination.^3^ The persistence of bacteria on surfaces and within complex microenvironments contributes significantly to treatment failure and disease transmission, as such settings can enhance tolerance to antimicrobial agents and host immune responses. These challenges represent a major barrier to effective infection control and continue to intensify the global AMR crisis. In the United Kingdom alone, chronic and recurrent bacterial infections are estimated to cost approximately £2 billion per year to the National Health Service (NHS), motivating sustained investment in research aimed at improving detection, monitoring, and intervention strategies.^4,5^ Beyond healthcare, bacterial colonisation of industrial and environmental surfaces—such as pipelines, water distribution systems, and marine vessels—leads to biofouling, corrosion, and energy losses, collectively accounting for multi-billion-pound annual losses worldwide.^6,7^

To design timely and effective strategies to mitigate the detrimental effects of surface-associated bacterial contamination, it is essential to understand the fundamental mechanisms governing microbial survival, growth, and interaction in confined environments. However, bacterial systems are inherently dynamic and complex, making their monitoring and prediction—particularly for control purposes—a major analytical challenge. This complexity is further amplified in multispecies settings, where interactions between different bacterial species and their spatial organisation within micron-scale surface-associated microenvironments strongly influence collective behaviour and functional outcomes, adding additional layers of difficulty to experimental analysis.^8–10^

Fluorescence-based microscopy remains the most widely used approach for visualising and analysing multispecies bacterial systems on surfaces, with confocal laser scanning microscopy (CLSM) often considered the gold standard due to its high spatial and temporal resolution, enabling quantification of population distribution, spatial organisation, and local biomass.^11–13^ To distinguish bacterial populations, researchers frequently employ fluorescent protein (FP) tagging, whereby cells are genetically engineered to express reporter proteins with distinct emission spectra.^14–16^ This multicolour labelling allows real-time visualisation of spatial organisation and interspecies interactions within mixed bacterial assemblies. However, studies have shown that FP expression can impose a metabolic burden, resulting in alterations to bacterial physiology and phenotype. For instance, *Pseudomonas chlororaphis* expressing GFP showed altered pigmentation and complete loss of antifungal metabolite production, such as phenazines and pyrrolnitrin, resulting in abrogation of biocontrol activity against *Pythium ultimum*.^17^ In *Vibrio aestuarianus*, GFP-tagging of a weakly pathogenic strain unexpectedly increased its virulence.^18^ Conversely, in a study comparing two *Vibrio anguillarum* strains, GFP expression was found to negatively affect growth and swimming motility in one strain, while in the other, only motility was impaired. These physiological perturbations may influence microbial behaviour and interspecies interactions, raising concerns about whether FP-tagged strains accurately represent their wild-type counterparts under experimentally relevant conditions. Understanding the biochemical and physiological consequences of FP tagging is therefore essential when interpreting data from mixed-species and surface-associated bacterial systems.

Vibrational spectroscopic techniques, including Raman and infrared (IR) spectroscopy, offer powerful, label-free approaches for characterising bacterial systems. Both techniques provide rich biochemical fingerprints that reflect the global molecular composition of cells, enabling simultaneous detection of lipids, proteins, nucleic acids, carbohydrates, and other important biomolecules.^19,20^ These methods have been successfully applied to bacterial studies including bacterial typing,^21–23^ antimicrobial resistance,^24^ and microbial metabolite profiling.^25^ Given their ability to provide holistic biochemical information in a label-free manner, Raman and IR spectroscopy offer ideal tools for studying the effects of FP-tagging on bacterial biochemistry. To date, only one study has directly examined the biochemical consequences of eGFP expression in *Escherichia coli* using FTIR spectroscopy, which combined FTIR metabolic fingerprinting with gas chromatography-mass spectrometry (GC-MS) to evaluate the metabolic burden of recombinant eGFP production.^26^ Building on this foundation, the present study employs both Raman and infrared spectroscopies to assess how FP-tagging influences bacterial biochemistry. Two of the most widely-studied and well-characterised bacterial species, Gram-positive *Staphylococcus aureus* and Gram-negative *Pseudomonas aeruginosa*, were selected as the vibrational spectral profiles of their wild-type have been reported. The spectroscopic signatures of these were compared with their fluorescent counterparts expressing distinct FPs, eGFP and mCherry.

## Experimental

### Bacterial strains and growth conditions

The bacterial strains used in this study were *Pseudomonas aeruginosa* PAO1-L as Gram-negative species and *Staphylococcus aureus* SH1000 as Gram-positive, along with their modified counterparts containing a plasmid expressing fluorescent reporter proteins mCherry (*P. aeruginosa* PAO1-L mCherry) and eGFP (*S. aureus* SH1000 eGFP) provided by the University of Nottingham. All strains were cultured on Tryptic Soy agar plates and incubated at 37 °C for 24 h. Following incubation, 3–4 bacterial colonies were harvested from the surface of each plate using sterile inoculating loops and resuspended in 1 mL of deionised water. Subsequently, 10 µL aliquots of each suspension were deposited onto CaF_2_ substrates and allowed to air-dry at room temperature.

### FTIR spectroscopy

FTIR spectra of bacterial samples were collected in transmission mode using a Bruker Lumos II microscope in the 4000–600 cm^−1^ range at a resolution of 4 cm^−1^, with 64 spectra co-added and averaged to improve the signal-to-noise ratio. Spectral datasets were acquired in imaging mode generating hyperspectral maps containing 1024 spectra, each representing a pixel from the detector. Background scans were obtained from a region outside the sample field.

### Raman spectroscopy

Raman spectra were measured using a Renishaw inVia Raman microscope (Renishaw Plc., Gloucestershire, UK) equipped with a 532 nm diode laser and air-cooled CCD detector. All spectra were acquired using the laser power adjusted on the sample to ∼30 mW, 10–20 s exposure time, three accumulations, and 600 lines per mm grating resulting in a spectral resolution of 6 cm^−1^. Instrument control and data capture were achieved using the GRAMS WiRE 3.4 software (Galactic Industries Corp. Salem, NH), and a 50× magnifying objective was used for sample observation.

### Data analysis

All collected FTIR and Raman spectra were processed using MATLAB software version 2021a (The Mathworks Inc., Natwick, USA). Spectral datasets were pre-processed (baseline correction, vector normalisation) and subjected to principal component analysis (PCA).

## Results and discussion

### FTIR spectroscopy

**Fig. 1:**
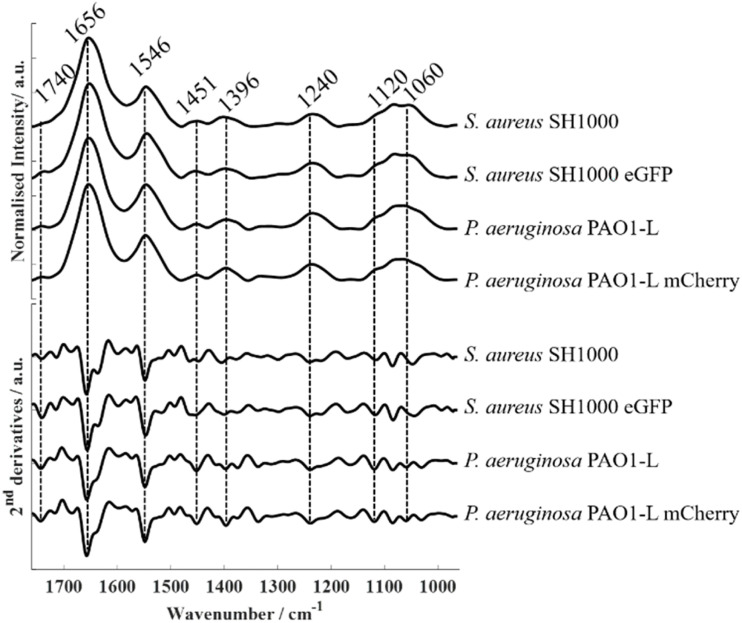
Fourier transform infrared (FTIR) spectra and corresponding second-derivative spectra of *S. aureus* SH1000, *S. aureus* SH1000 eGFP, *P. aeruginosa* PAO1-L, and *P. aeruginosa* PAO1-L mCherry.

**Fig. 2:**
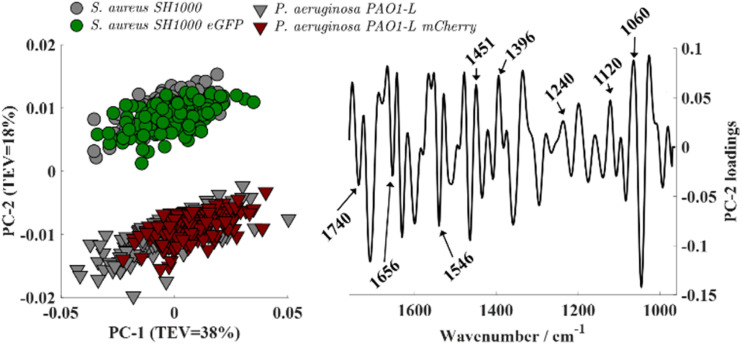
Principal component analysis (PCA) applied to second derivatives of wild-type and fluorescent-protein-expressing bacterial strains. Left: PCA scores plot based on the 1800–900 cm^−1^ spectral region showing clear separation between bacterial species. Right: Corresponding PC-2 loadings.

FTIR spectroscopy is a powerful analytical technique for probing biological samples, including bacterial systems, as it provides a comprehensive biochemical snapshot that reflects the overall biochemistry of the organism at a given time. Fig. 1 shows representative FTIR spectra (top) acquired from *S. aureus* SH1000, *S. aureus* SH1000-eGFP, *P. aeruginosa* PAO1-L, and *P. aeruginosa* PAO1-L-mCherry. To enhance interpretability, second-derivatives were calculated (Fig. 1, bottom) from the spectra as this approach effectively minimizes the contribution of baseline distortions, improves spectral resolution, and uncovers overlapping sub-bands that are not discernible in the raw spectra.^27^ The absorption bands observed in the spectral datasets acquired from bacterial species employed in this study are consistent with typical bacterial biochemical fingerprints. The band at approximately 1740 cm^−1^ corresponds to the C=O stretching vibration of carbonyl ester functional groups from lipids.^28^ The prominent amide I (∼1656 cm^−1^) and amide II (∼1546 cm^−1^) bands arise from protein backbone vibrations (C=O stretching and N–H bending/C–N stretching, respectively).^29^ The band peaking at 1451 cm^−1^ is attributed to CH_2_/CH_3_ groups in branched lipids,^22,28^ while 1396 cm^−1^ is due to symmetric stretching of carboxylate groups from (–COOH) fatty acids and amino acid side chains.^22,28^ The band around 1240 cm^−1^ corresponds to phosphate-containing compounds (PO_2_^−^) such as nucleic acids and phospholipids,^22,28^ while those at 1120 and 1060 cm^−1^ are assigned to C–O–C symmetric stretching of polysaccharides.^28^ Second-derivatives computed from spectra were subjected to principal component analysis (PCA). The resulting scores plot (Fig. 2, left) showed clear species-level discrimination along the PC-2 axis, with both *S. aureus* strains clustering on the positive side and both *P. aeruginosa* strains grouping on the negative side. Intra-group variability was observed, reflected by the dispersion of replicate scores within the same class. This reduced data reproducibility likely arises from minor differences in sample preparation such as variations in film thickness, drying effects, or growth media residuals, as well as from inherent physiological heterogeneity among biological replicates.

Analysis of the PC-2 loadings revealed that the separation of bacterial strains along this component primarily reflects the fundamental biochemical differences between Gram-positive and Gram-negative species, arising from their distinct cell wall and membrane architectures. These structural variations result in different relative abundances of key biomolecular constituents—polysaccharides, phosphates, lipids, and proteins—which in turn shape their FTIR spectral fingerprints. Gram-positive bacteria such as *S. aureus* possess a thick peptidoglycan layer (constituting up to 40–80% of the cell wall dry weight) enriched with teichoic acids—unique polyalcohol phosphates—that contribute to a higher phosphate content.^30^ Accordingly, *S. aureus* displayed stronger loading contributions from carbohydrate and phosphate-associated vibrations in the 1200–900 cm^−1^ region. In contrast, Gram-negative bacteria such as *P. aeruginosa* have a much thinner peptidoglycan layer and lack teichoic acids, instead featuring an outer membrane rich in phospholipids and proteins.^30^ This was reflected in the PC-2 loadings by dominant lipid and protein bands characterized by more intense amide and lipid absorptions in the 1700–1500 cm^−1^ range corresponding to its double-membrane architecture.

Interestingly, no separation was observed between wild-type and fluorescent-protein-expressing strains, as their scores consistently overlapped within each species. Analysis of higher-order principal components revealed no additional segregation. Also, pairwise PCA comparisons (*S. aureus* SH1000 *vs.* SH1000-eGFP; *P. aeruginosa* PAO1-L *vs.* PAO1-L-mCherry) also showed overlapping distributions (Fig. S1). These results indicate that the expression of fluorescent proteins (eGFP or mCherry) did not cause detectable biochemical alterations at the whole-cell level as assessed by FTIR spectroscopy.

Spectral readings from wild-type and fluorescent-protein-expressing strains showed no additional absorption bands or measurable peak shifts. Although the labelled strains express heterologous fluorescent proteins, their vibrational signatures largely overlap with those of native proteins within the amide I and II regions. While FTIR spectroscopy provides a holistic biochemical fingerprint by probing the vibrational motions of molecular bonds in molecules, discriminating specific molecular species within the same class (*e.g.*, identifying which protein or component is altered) remains challenging due to overlapping absorption features within the infrared range.

### Raman spectroscopy

**Fig. 3:**
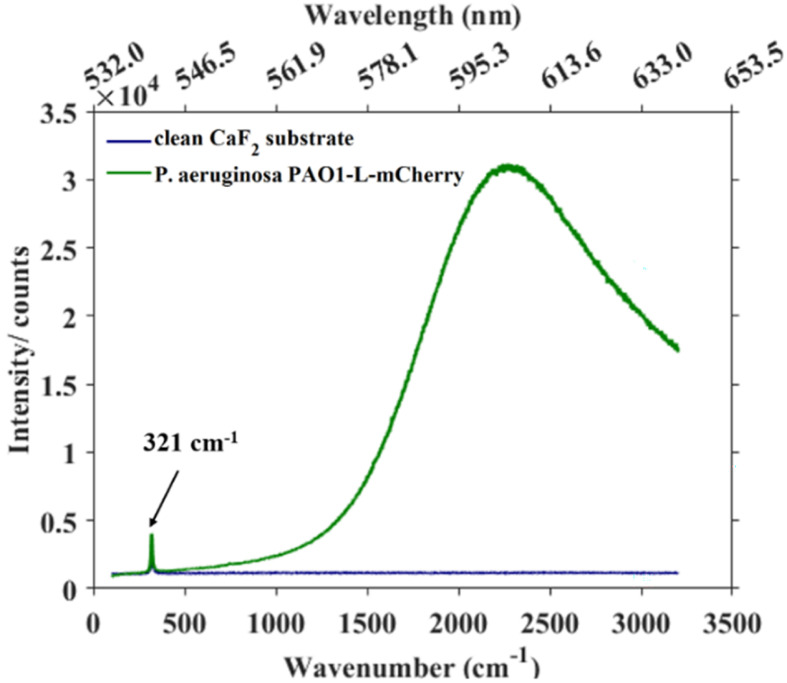
Raman spectra acquired using reduced laser power and integration time to avoid detector saturation, showing the spectra of a clean CaF_2_ substrate and *P. aeruginosa* PAO1-L-mCherry on CaF_2_. The sharp peak at 321 cm^−1^ is a substrate-related contribution from CaF_2_.

The presence of fluorescent proteins in a cell opens the option for Raman spectroscopy to operate either in fluorescence-inducing or fluorescence-free conditions. We illustrate both approaches here. In this study, a 532 nm laser was used as the excitation source, providing photons whose energy coincides with the electronic transition of the mCherry fluorophore. As a result, *P. aeruginosa* strains expressing mCherry produced an intense fluorescence background that masked Raman peaks corresponding to biochemical components from bacteria. By setting the experimental parameters to avoid saturation in the detector (*i.e.*, lowering the laser power and integration time) and scanning the 100–3200 cm^−1^ spectral range of *P. aeruginosa* PAO1-L-mCherry, the resulting spectrum clearly shows an intense and broad band around 2200 cm^−1^ (Fig. 3) representing the mCherry fluorescent photons. Additionally, a sharp peak at 321 cm^−1^ can also be observed, corresponding to the CaF_2_ substrate. Although, the fluorescence background masks the biochemical information, the inherent strength of the fluorescent response would enable FP-containing cells within a population to be detected at the single-cell levels. This fluorescence interference is intrinsic to Raman spectroscopy but does not affect FTIR measurements, as infrared photons lack sufficient energy to induce electronic transitions.

**Fig. 4:**
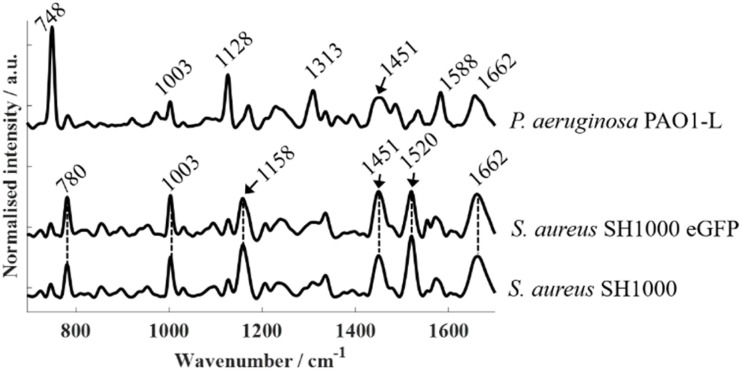
Raman spectra of *S. aureus* SH1000, *S. aureus* SH1000 eGFP, and *P. aeruginosa* PAO1-L.

Fig. 4 presents Raman spectra acquired from fluorescence-free strains using 532 nm excitation: *S. aureus* SH1000, *S. aureus* SH1000-eGFP, and *P. aeruginosa* PAO1-L. Compared to FTIR spectroscopy, which yielded broadly similar band patterns across all bacterial strains, Raman spectra revealed pronounced species-specific differences between *P. aeruginosa* and *S. aureus* that can be identified even without advanced statistical analysis. While *S. aureus* SH1000 and *S. aureus* SH1000-eGFP exhibited nearly identical spectral profiles, both were clearly differentiated from *P. aeruginosa* PAO1-L. Across all strains, a few common bands were detected—most notably at ∼1003 cm^−1^ (phenylalanine ring breathing), ∼1451 cm^−1^ (CH_2_/CH_3_ bending from lipids and proteins), and ∼1662 cm^−1^ (amide I, C=O stretching).^22,31^ However, species-specific peaks dominated the overall distinction. In *S. aureus*, strong carotenoid-related bands were observed at ∼1158 cm^−1^ and ∼1520 cm^−1^, arising from C–C and C=C stretching in the polyene chain, respectively, along with a weaker contribution around ∼1003 cm^−1^ attributable to C–CH_3_ deformations that overlap with the phenylalanine band. These features correspond to staphyloxanthin, the carotenoid pigment responsible for the characteristic golden coloration of *S. aureus*.^31^ A band peaking at 1554 cm^−1^ can be observed in *S. aureus* SH1000-eGFP, however, this vibrational mode is attributed to O_2_ instead of a molecular component within bacterial biochemistry. In contrast, *P. aeruginosa* lacked carotenoid bands but exhibited prominent cytochrome-related peaks at 748 cm^−1^ (pyrrole breathing), 1128 cm^−1^ (C–N stretching), 1313 cm^−1^ (CH_3_/CH_2_ deformation), and 1588 cm^−1^ (C=C stretching), consistent with previously reported cytochrome signatures.^22^

The pronounced intensity of carotenoid and cytochrome peaks in the Raman spectra arises primarily from enhancement effects. These molecules possess conjugated π-electron systems that confer strong polarizability, and under resonance or pre-resonance conditions, their vibrational modes are significantly intensified.^32^ In this study, the 532 nm excitation laser coincides with the electronic transitions of both carotenoids and cytochromes, thereby inducing strong resonance Raman enhancement. As a result, even at relatively low intracellular concentrations, these molecules produced dominant Raman signals that stood out against the spectral background. By contrast, FTIR spectroscopy does not benefit from resonance effects, explaining the absence of pigment-related features in the infrared spectra.

Beyond the clear differentiation observed between *S. aureus* and *P. aeruginosa*, the distinctive pigment-associated Raman features highlight the potential of Raman spectroscopy as a label-free technique for monitoring bacterial composition and metabolic dynamics in mixed-species and surface-associated systems. Carotenoids and cytochromes, which are naturally produced by many bacterial species, give rise to resonance-enhanced Raman bands that can serve as intrinsic biochemical markers for tracking population changes or metabolic activity without the need for fluorescent labelling. This capability enables longitudinal, non-destructive analysis of bacterial communities under native conditions while avoiding potential artefacts associated with fluorescent protein expression. However, conventional Raman spectroscopy presents limitations compared with fluorescence imaging, particularly in spatial and temporal resolution. The inherently weak scattering cross-section of most biomolecules results in relatively long acquisition times, restricting its applicability for rapid three-dimensional imaging. Therefore, resonance enhancements of the type observed here offer a route towards detection at increasing spatial resolution. Recent advances in stimulated Raman scattering (SRS) microscopy have also begun to overcome these challenges by offering significantly improved sensitivity and imaging speed.^33^ SRS has been successfully applied to microbial systems such as yeast^34^ and single-species bacterial cultures,^35^ yet, to date, no study has explored its application for monitoring mixed-species bacterial systems in surface-associated environments.

**Fig. 5:**
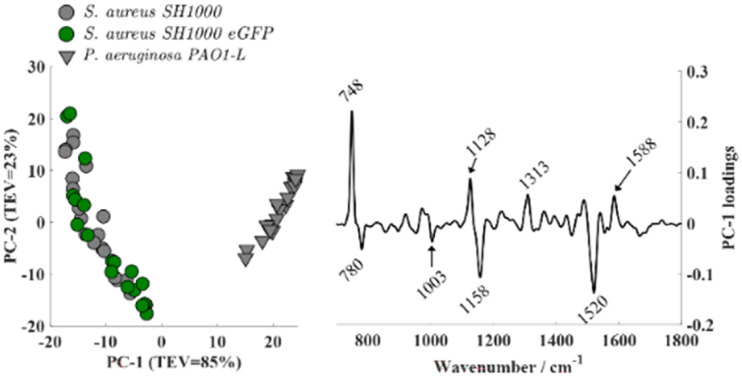
Principal component analysis (PCA) applied to Raman spectra of *S. aureus* SH1000, *S. aureus* SH1000 eGFP, and *P. aeruginosa* PAO1-L.

PCA applied to Raman spectra (Fig. 5) revealed clear species-level clustering. *S. aureus* (both wild-type and eGFP-expressing) separated distinctly from *P. aeruginosa*, with loadings dominated by the characteristic carotenoid bands at 1520, 1158, and 1003 cm^−1^ in *S. aureus* as well as cytochrome-associated bands at 1588, 1313, 1128, and 748 cm^−1^ in *P. aeruginosa*. Consistent with the FTIR results, PCA scores comparing *S. aureus* SH1000 and *S. aureus* SH1000-eGFP exhibited substantial overlap (Fig. S2), indicating no discernible biochemical differences between the wild-type and fluorescent strains. Analysis of higher-order principal components such as PC2 *vs.* PC3 revealed some discrimination among datasets, however, the loadings analysis confirmed that these were mainly due to variations in O_2_ levels during the measurements.

## Conclusions

This study provides the first combined FTIR and Raman spectroscopic evaluation of the biochemical consequences of fluorescent-protein tagging in *S. aureus* SH1000 and *P. aeruginosa* PAO1-L. Overall, FP expression (eGFP or mCherry) did not produce additional absorption bands, Raman peaks, or PCA-detectable segregation relative to the wild-type strains, indicating that the introduction of fluorescent proteins does not measurably alter the global biochemical composition of these bacteria under the conditions tested. FTIR spectroscopy robustly distinguished Gram-positive from Gram-negative species through differences in cell wall and membrane composition, while Raman spectroscopy revealed strong species-specific pigment signatures arising from resonance enhancement of carotenoids in *S. aureus* and cytochromes in *P. aeruginosa*. Collectively, these findings support the biochemical fidelity of fluorescently labelled bacterial strains and highlight the complementary roles of FTIR and Raman spectroscopy in future correlative studies of bacterial physiology and composition in surface-associated and mixed-species system.

## Author contributions

C. L. contributed towards experimental design, investigation, data analysis, data interpretation and writing the original draft. J. A. contributed towards sample preparation. R. R. contributed towards conceptualisation, funding acquisition, data interpretation and supervision. All authors contributed towards reviewing and editing the writing.

## Conflicts of interest

There are no conflicts to declare.

## Supplementary Material

FD-265-D5FD00175G-s001

## Data Availability

Data for this article are available at Zenodo at https://doi.org/10.5281/zenodo.18234350. Supplementary information (SI): additional principal component analysis (PCA) results, including pairwise comparisons of wild-type and fluorescent-protein-expressing strains, along with supporting figures (Fig. S1 and S2). See DOI: https://doi.org/10.1039/d5fd00175g.
