# Supplementary material for: Vibrational spectroscopic characterisation of fluorescent-protein-tagged and wild-type bacteria in surface-associated microdroplets
Source: Faraday Discuss. 2026 Apr 20. Online ahead of print. doi: 10.1039/d5fd00175g (PMC13094341; doi:10.1039/d5fd00175g)
Supplement: FD-OLF-D5FD00175G-s001 [file FD-OLF-D5FD00175G-s001.pdf]

Faraday Discussions

PAPER

Vibrational Spectroscopic Characterisation of Fluorescent-Protein-Tagged and Wild-Type Bacteria in Surface-Associated Microdroplets

[a.] Open Innovation Hub for Antimicrobial Surfaces, Surface Science Research Centre, Department of Chemistry, University of Liverpool, Liverpool L69 3BX, United Kingdom

Cassio Lima,[a] Jontana Allkja,[a] and Rasmita Raval*[a]

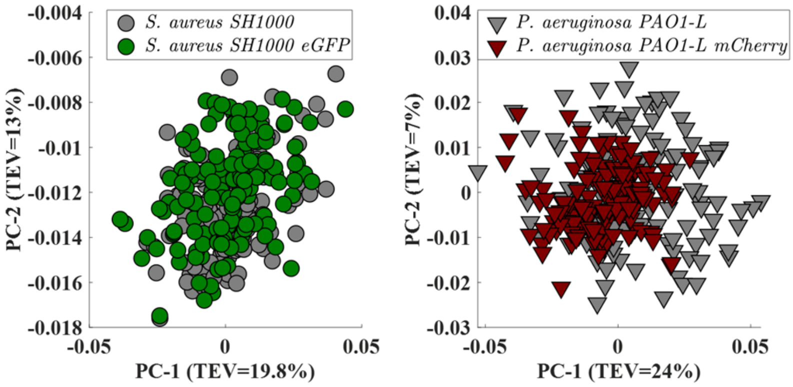

Figure S1. Principal component analysis (PCA) applied to second derivatives of wild-type and fluorescent-protein-expressing bacterial strains. PCA scores obtained for *S. aureus* SH1000 *vs. S. aureus* SH1000eGFP **(left)** and *P. aeruginosa* PAO1-L *vs. P. aeruginosa* PAO1-L mCherry **(right)**.

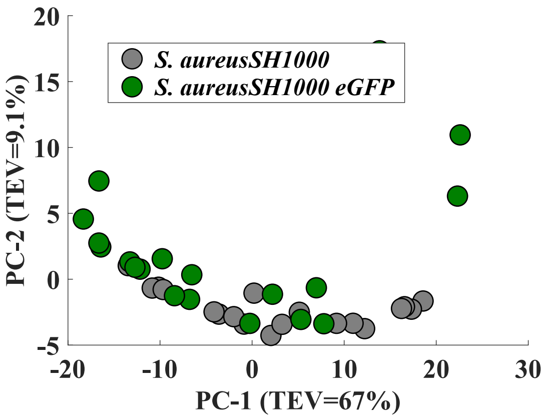

Figure S2. Principal component analysis (PCA) applied to Raman spectra of *S. aureus* SH1000 *vs. S. aureus* SH1000 eGFP
